# Machine learning for differentiating between pancreatobiliary-type and intestinal-type periampullary carcinomas based on CT imaging and clinical findings

**DOI:** 10.1007/s00261-023-04151-1

**Published:** 2024-01-18

**Authors:** Tao Chen, Danbin Zhang, Shaoqing Chen, Juan Lu, Qinger Guo, Shuyang Cai, Hong Yang, Ruixuan Wang, Ziyao Hu, Yang Chen

**Affiliations:** 1 Department of Radiology, The First Affiliated Hospital, Zhejiang University School of Medicine, 79 Qingchun Road, Hangzhou, 310003 Zhejiang China; 2 Department of Radiology, The Second Affiliated Hospital and Yuying Children’s Hospital of Wenzhou Medical University, 109 Xueyuan West Road, Wenzhou, 325027 Zhejiang China; 3 Liverpool Centre for Cardiovascular Science at University of Liverpool, Liverpool John Moores University and Liverpool Heart and Chest Hospital, William Henry Duncan Building, 6 West Derby St, Liverpool, Merseyside L7 8TX UK; 4 Department of Computer Science and Software Engineering, The University of Western Australia, Crawley, WA 6009 Australia; 5 School of Medicine, The University of Western Australia, Crawley, WA 6009 Australia; 6 Harry Perkins Institute of Medical Research, Murdoch, WA 6150 Australia; 7 School of Electronics and Computer Science, University of Liverpool, Brownlow Hill, Liverpool, Merseyside L69 3BX UK

**Keywords:** Periampullary adenocarcinoma, X-ray computed tomography, Histopathology, Prediction model, Machine learning

## Abstract

**Purpose:**

To develop a diagnostic model for distinguishing pancreatobiliary-type and intestinal-type periampullary adenocarcinomas using preoperative contrast-enhanced computed tomography (CT) findings combined with clinical characteristics.

**Methods:**

This retrospective study included 140 patients with periampullary adenocarcinoma who underwent preoperative enhanced CT, including pancreaticobiliary (*N* = 100) and intestinal (*N* = 40) types. They were randomly assigned to the training or internal validation set in an 8:2 ratio. Additionally, an independent external cohort of 28 patients was enrolled. Various CT features of the periampullary region were evaluated and data from clinical and laboratory tests were collected. Five machine learning classifiers were developed to identify the histologic type of periampullary adenocarcinoma, including logistic regression, random forest, multi-layer perceptron, light gradient boosting, and eXtreme gradient boosting (XGBoost).

**Results:**

All machine learning classifiers except multi-layer perceptron used achieved good performance in distinguishing pancreatobiliary-type and intestinal-type adenocarcinomas, with the area under the curve (AUC) ranging from 0.75 to 0.98. The AUC values of the XGBoost classifier in the training set, internal validation set and external validation set are 0.98, 0.89 and 0.84 respectively. The enhancement degree of tumor, the growth pattern of tumor, and carbohydrate antigen 19–9 were the most important factors in the model.

**Conclusion:**

Machine learning models combining CT with clinical features can serve as a noninvasive tool to differentiate the histological subtypes of periampullary adenocarcinoma, in particular using the XGBoost classifier.

**Supplementary Information:**

The online version contains supplementary material available at 10.1007/s00261-023-04151-1.

## Introduction

Periampullary adenocarcinoma is defined as a collective term for several malignant tumors occurring within 2 cm of the duodenal papilla with the traditional anatomical subtypes including pancreatic cancer, ampulla cancer, bile duct cancer, and duodenal cancer [1]. Although the optimal treatment is curative resection [2, 3], there are significant differences in survival rates between patients with each subtype of tumors, with duodenal cancer having the highest 5-year survival rate (59%) and pancreatic cancer having the lowest (15%) [4]. However, the ampulla region is small and complex, which makes it difficult to distinguish anatomically.

Histologically, most periampullary adenocarcinomas can be divided into intestinal and pancreaticobiliary types [5]. A growing number of studies have emphasized that histological phenotype of periampullary adenocarcinoma plays an important role in cancer progression and therapeutic response [5, 6]. Pancreatobiliary periampullary adenocarcinoma is more aggressive and has a worse prognosis than intestinal periampullary adenocarcinoma, suggesting that histopathological phenotype is an independent predictor of survival in patients with periampullary adenocarcinoma [7–9]. Furthermore, pancreatobiliary- and intestinal-type periampullary adenocarcinoma respond differently to various chemotherapeutic agents, and early histological typing can help predict the efficacy of adjuvant chemotherapeutic agents [6, 10, 11]. Findings in previous studies have shown that pancreaticobiliary type is associated with shorter survival as well as earlier recurrence[5, 12–15]. Currently, the gold standard for diagnosis histological subtypes of periampullary adenocarcinoma is postoperative immunohistochemical staining markers, e.g., mucin1, mucin 2, cytokeratin7, cytokeratin17, and so on. [16]. Therefore, noninvasive biomarkers for preoperative prediction of histological subtypes are needed to improve periampullary adenocarcinoma treatment.

Computed tomography (CT) and magnetic resonance imaging (MRI) are widely used in clinical practice for the diagnosis, staging, and resectability assessment of periampullary adenocarcinoma. MRI has higher soft tissue resolution than CT, giving it an advantage in determining the anatomical relationships between periampullary adenocarcinoma and adjacent structures; however, it is limited by high cost, time consuming, and susceptibility to motion interference. CT is often used more extensively to evaluate the ampullary abnormalities due to the advantages of ease of acquisition, low cost, and high spatial resolution. The association between imaging features and the histological phenotype of periampullary adenocarcinoma has gained increasing attention [17–19]. However, the value of dynamic contract-enhanced CT in differentiating pancreatobiliary and intestinal-type periampullary adenocarcinoma is unclear. Additionally, recent studies have shown the diagnostic value of serum carbohydrate antigen 19–9 (CA19-9) and total bilirubin levels in differentiating benign and malignant ampullary adenocarcinoma [20].

Considering the extensive application of machine learning (ML) classifiers in the medical field, we aimed to construct models based on dynamic contract-enhanced CT and clinical features using various ML classifiers to differentiate pancreatobiliary- and intestinal-type periampullary adenocarcinomas, thereby helping clinicians predict the prognosis and guide the treatment for patients with periampullary adenocarcinoma.

## Methods

### Study population

Surgical pathology records were searched in pathology database using the terms "periampullary adenocarcinoma," "lower part of common bile duct," "duodenal papilla," or "Vater" in combination with "pancreaticobiliary" or "intestinal." The inclusion criteria were as follows: (1) patients diagnosed surgically with periampullary adenocarcinoma and histologically confirmed pancreatobiliary- or intestinal-type periampullary adenocarcinoma; (2) standard unenhanced and contrast-enhanced abdominal CT imaging performed < 30 days before surgical resection; and (3) no preoperative invasive therapy. We excluded patients using the following criteria: (1) histologically confirmed mixed pancreatobiliary and intestinal types; (2) patients with other synchronous malignant neoplasms or received previous anticancer treatment; and (3) tumor lesions could not be identified on CT.

**Fig. 1:**
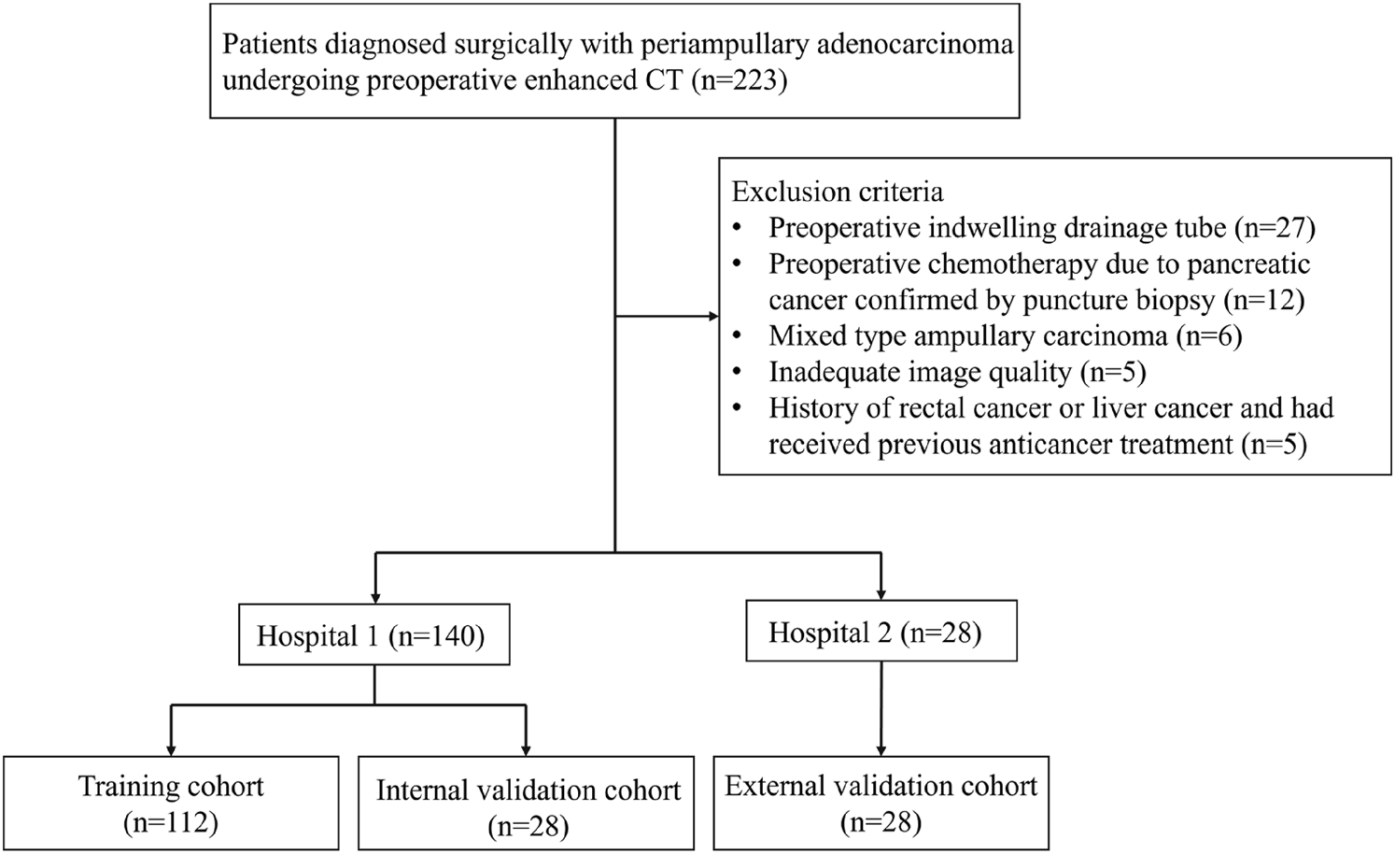
Flowchart of the study cohort

This was a multicenter retrospective study that included two hospitals and was approved by the institutional review board of the two hospitals and patient informed consent was waived. The data from Hospital one would be randomly assigned to the training cohort and the internal validation set in a ratio of 8:2, and the data from Hospital two would be the external validation set (Fig. 1). A total of 187 patients from January 2019 to December 2022 were enrolled from Hospital one. Among them, 23 patients underwent preoperative indwelling drainage tube, 10 patients received preoperative chemotherapy due to pancreatic cancer confirmed by puncture biopsy, 4 patients had mixed-type ampullary adenocarcinoma, 5 patients had unsatisfactory image quality assessment due to motion artifacts, and 5 patients who had history of rectal cancer or liver cancer and received previous anticancer treatment were excluded from the study. Consequently, 140 patients were enrolled in the study (Fig. 1).

We recruited patients between November 2013 and December 2022 from Hospital two to form an external validation dataset. This dataset yielded 36 patients. Among them, 4 patients underwent preoperative indwelling drainage tube, 2 patients received preoperative chemotherapy due to pancreatic cancer confirmed by puncture biopsy, and 2 patients who had mixed-type ampullary adenocarcinoma were excluded from the study. Consequently, 28 patients were included in the study (Fig. 1).

### Clinical data collection

The patient clinical information, including age, sex, history of cholecystectomy, serum total bilirubin, and CA19-9 levels were retrieved from medical records. The recorded laboratory data must be measured within 2 weeks before CT examination. Elevated serum total bilirubin and CA19-9 were defined as total bilirubin ≥ 21 μmol/L and CA19-9 ≥ 37 U/mL.

### CT image acquisition

Patients were required to fast for at least 4 h and drink at least 500 ml of pure water as an oral contrast agent to fill the duodenum cavity before the CT examination. Images from Hospital one were acquired using three CT scanners: a 16-slice canner (Aquilion; Toshiba Medical Systems, Tokyo, Japan), 64-slice scanner (LightSpeed VCT, GE Medical Systems, Milwaukee, USA), and a 256-slice scanner (Brilliance iCT; Philips Healthcare, Cleveland, OH, USA). The CT parameters were tube voltage of 100 or 120 kVp, tube current of 200–700 mAs, pixel spacing of 0.539–0.881 mm, and slice thickness of 0.625–5.000 mm (median slice thickness, 1 mm). The nonionic contrast agents used were Iohexol (Yangtze River Pharmaceutical Group, Taizhou, China) and Iodixanol and Iohexol (GE Healthcare Ireland, Carrigtohill, Ireland). The high-pressure syringe (3.0 mL/s) was used to inject nonionic contrast agent (1.5 mL/kg). After unenhanced scanning, arterial phase (25–35 s), portal venous phase (55–75 s), and delayed phase (120–180 s) were performed.

Images from Hospital two were acquired using two CT scanners: a 16-slice canner (Lightspeed; GE) and a 16-slice canner (Somatom Emotion; Siemens). The CT parameters were tube voltage of 120 kVp and tube current of 300 mAs. The layer thickness is 1 mm, and the layer spacing is 1 mm. The nonionic iodine contrast agent Ioferol (300 mgI/mL) was injected through the cubical vein with a total volume of 80–100 mL and an injection flow rate of 2.5–3.0 mL/s with a high-pressure syringe. After unenhanced scanning, arterial phase (25 s), portal venous phase (50–60 s), and delayed phase (150–180 s) were performed.

### Image analysis

All images were reviewed by two radiologists independently (C.T. and C.S., with 6 and 7 years of experience in abdominal CT interpretation, respectively). Both observers were blinded to the clinical and histopathological data but knew the patients had periampullary adenocarcinoma. Any discrepancy was resolved by a third radiologist (Y.H. with 25 years of experience in abdominal CT interpretation). Throughout the whole evaluation process, the radiologist needs to evaluate the CT images comprehensively in the transverse, sagittal, and coronal views, as well as evaluating the CT images in all phases in order to make a final conclusion.

The evaluation of imaging features was referred to previous relevant studies [21–23]. The qualitative evaluation features were as follows: (a) presence of ampulla mass; (b) growth pattern (intrinsic, extrinsic, or mixed); (c) presence of bulging ampulla; (d) shape of distal common bile duct (CBD) margin (smooth or irregular); (e) symmetry of distal CBD lumen (symmetric or asymmetric); (f) pattern of distal CBD narrowing (gradual tapering or abrupt narrowing); (g) pattern of biliary dilatation (central or proportional); (h) presence of intrahepatic ducts (IHD) dilatation; (i) IHD dilatation extent (no to mild dilation or moderate to severe dilation); (j) presence of thickened distal CBD wall (> 1.5 mm); (k) presence of dilated main pancreatic duct (MPD) (> 3 mm); (l) presence of peripancreatic lymph node enlargement (short diameter > 1 cm); (m) presence of necrosis, calcification, or cystic within the lesion; (n) enhancement degree (similar or different enhancement degree to adjacent normal duodenal wall on the portal venous phase); (o) peak enhancement phase of lesion (arterial phase, portal venous phase or delayed phase); (p) presence of main vascular involvement; (q) presence of periampullary duodenal diverticulum; and (r) presence of pancreatic involvement.

The quantitative evaluation features were as follows: (a) size of ampullary mass (mm); (b) angle of distal CBD end (°); (c) diameter of dilated CBD (mm); and (d) diameter of MPD (mm).

To confirm that the radiological features were highly reproducible and reliable, intraclass correlation coefficients (ICCs) were calculated to test interobserver agreement in the Hospital one dataset.

### Development and validation of prediction models

We used 18 qualitative CT features and 5 clinical variables to construct prediction models. To select the classifier prediction model with the highest discrimination between pancreatobiliary- and intestinal-type periampullary adenocarcinoma, we selected five ML classifiers including light gradient boosting (LightGBM), multi-layer perceptron (MLP), eXtreme Gradient Boosting (XGBoost), random forest, and logistic regression. We applied these classifiers in the training set and performed fivefold internal cross-validation to explore the optimal hyperparameters. Subsequently, the SHapley Additive exPlanations (SHAP) model interpretation method was used to individually calculate and analyze how each feature affected the output of the best classifier [24].

All models were validated in the internal validation cohort and external validation cohort. We plotted receiver operating characteristic curve (ROC) and compared the area under curve (AUC) using DeLong's test. Furthermore, we calculated the classifier’s accuracy, sensitivity, specificity, positive predictive value (PPV), negative predictive value (NPV), and F1 score. Finally, we selected the best predictive model based on metrics and plotted its calibration curve and clinical decision analysis (DCA) in external validation to assess their performance in clinical application.

### Evaluation of prognosis for periampullary adenocarcinoma

Prognostic analysis was performed in the Hospital one dataset (the training cohort and the internal validation cohort). Our prognostic outcomes included disease-free survival (DFS) and overall survival (OS), with DFS defined as the time from the start of tumor randomization to recurrence or death of the patient due to progression, and OS defined as the time to all-cause death. One patient was lost during follow-up.

### Statistical analysis

The tests of normality were conducted for continuous variables, using the Student’s *t*-test, expressed as mean ± standard deviation, for normally distributed continuous variables; using the Mann–Whitney *U* test, described as median with interquartile range, for non-normally distributed continuous variables. Categorical variables were expressed as percentage values using the chi-square or Fisher’s exact tests. Cohen’s kappa value and ICC values were calculated to evaluate the strength of interobserver agreement between the two radiologists (0.00–0.20 poor agreement, 0.21–0.40 fair agreement, 0.41–0.60 moderate agreement, 0.61–0.80 good agreement, and 0.81–1.00 excellent agreement). We used Kaplan–Meier curves to evaluate DFS and OS in the actual and predicted PAC subgroups to assess prognosis value of the model. The same survival analysis was performed based on the organ origin of the lesion, which was divided into four groups including the ampulla, duodenum, pancreas, and the CBD.

All statistical analyses were performed using SPSS software (version 27.0) and Python software (version 3.11). A two-tailed *P* value of < 0.05 indicates statistical significance.

## Results

### Patient characteristics

The overall study design is shown in Fig. 1. The demographic and pathological characteristics of the training cohort (*n* = 112), internal validation cohort (*n* = 28), and external validation cohort (*n* = 28) are listed in Table 1. Among the 168 patients (94 men, 74 women; mean age, 64.8 years; range, 29–89 years), 118 patients were the pancreatobiliary type (66 men, 52 women; mean age, 64.6 years; range, 29–89 years), and 50 patients were the intestinal type (28 men, 22 women; mean age, 65.1 years; range, 46–86 years).

**Table 1:** Characteristics of patients in each cohort

Clinical features	Training and internal validation cohorts (*n* = 140)			External validation cohort (*n* = 28)		
	Pancreatobiliary type (*n* = 100)	Intestinal type (*n* = 40)	*P* value	Pancreatobiliary type (*n* = 18)	Intestinal type (*n* = 10)	*P* value
Age, years (mean ± SD)	64.38 ± 10.36	65.50 ± 8.62	0.549	65.9 ± 8.32	63.5 ± 9.58	0.513
Male/female, *n*	58/42	23/17	0.957	8/10	5/5	0.778
Elevated CA19-9, *n* (%)	73 (73.0)	21 (52.5)	0.008	11 (61.1)	5 (50.0)	0.429
Elevated total bilirubin, *n* (%)	69 (69.0)	18 (45.0)	0.020	15 (83.3)	7 (70.0)	0.586
Post-cholecystectomy state, *n* (%)	9 (9.0)	4 (10.0)	0.854	1 (5.6)	0 (0)	0.466
Tumor differentiation, *n* (%)			0.068			0.466
Low	6 (6.0)	4 (10.0)		1 (5.6)	2 (20.0)	
Medium	92 (92.0)	32 (80.0)		13 (72.2)	6 (60.0)	
High	2 (2.0)	4 (10.0)		4 (22.2)	2 (20.0)	
Nodal involvement, *n* (%)	48 (48.0)	10 (25.0)	0.013	6 (33.3)	3 (30.0)	0.863
Perineural invasion, *n* (%)	83 (83.0)	13 (32.5)	< 0.001	10 (55.6)	5 (50.0)	0.790
Vessel involvement, *n* (%)	50 (50.0)	15 (37.5)	0.180	4 (22.2)	5 (50.0)	0.142
Tumor location			< 0.001			0.109
Duodenum	9 (9.0)	29 (72.5)		1 (5.6)	4 (40.0)	
Ampulla	28 (28.0)	6 (15.0)		8 (44.4)	3 (30.0)	
CBD	30 (30.0)	4 (10.0)		3 (16.7)	2 (20.0)	
Pancreas	33 (33.0)	1 (2.5)		6 (33.3)	1 (10.0)	

Data are expressed as mean ± SD, median (interquartile range), or *n* (%). Students’ *t*-test or Mann–Whitney *U*-test and Chi-square or Fisher’s exact test were used for comparisons among groups. Statistical significance (*P* < 0.05)

CBD, common bile duct

**Fig. 2:**
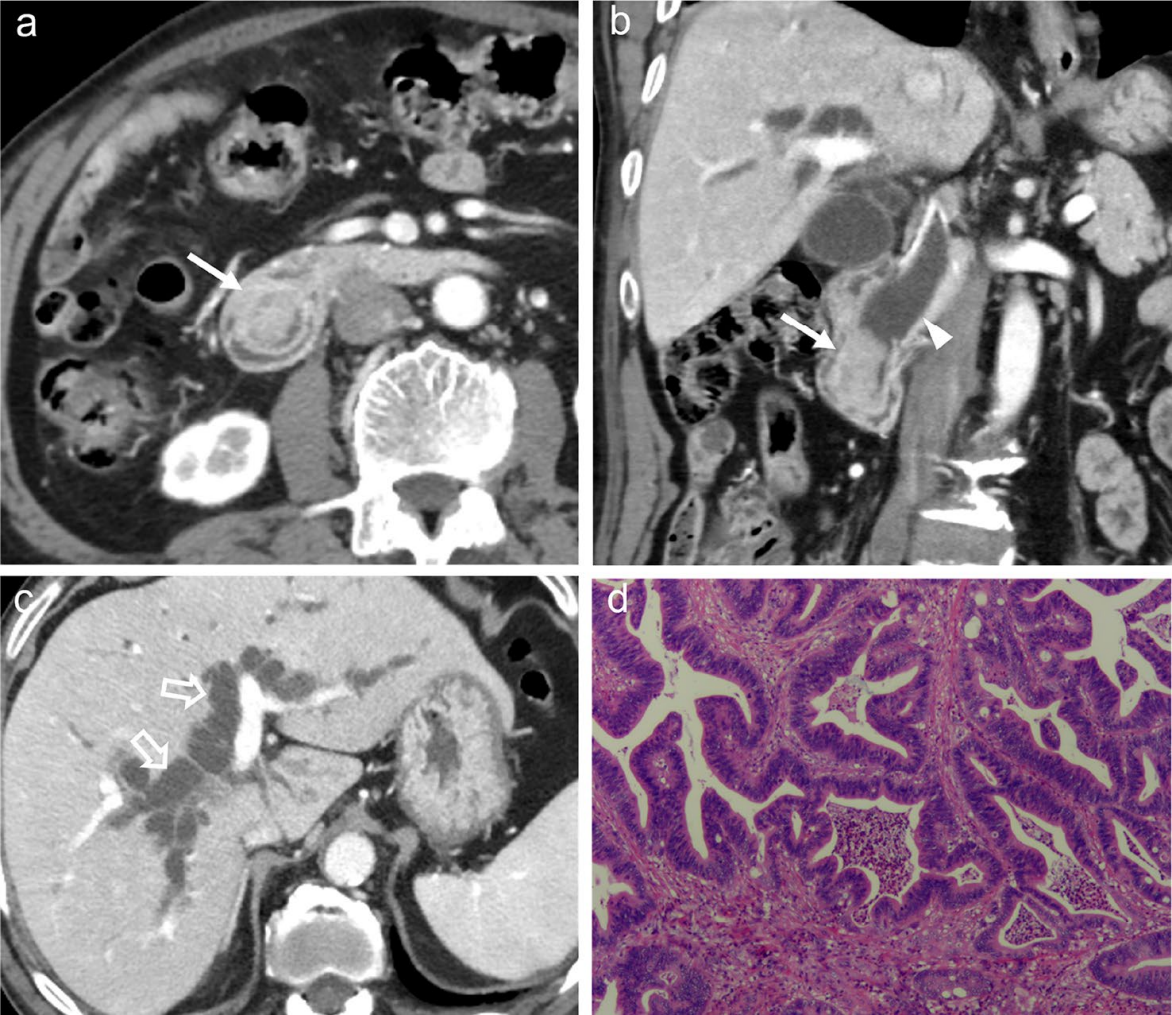
An example from a 72-year-old man who complained of epigastric pain and skin pruritus. Axial (**a, c**) and coronal reconstructed images (**b**) of enhanced CT in portal venous phase show a periampullary mass with enhancement degree similar to that of the duodenal wall (arrows), with an intrinsic growth pattern. The shape of distal CBD margin is smooth (arrowheads) accompanied by corresponding upstream bile duct dilatation (open arrow). The postoperative pathologic diagnosis of this patient was an intestinal-type periampullary adenocarcinoma (**d**)

**Fig. 3:**
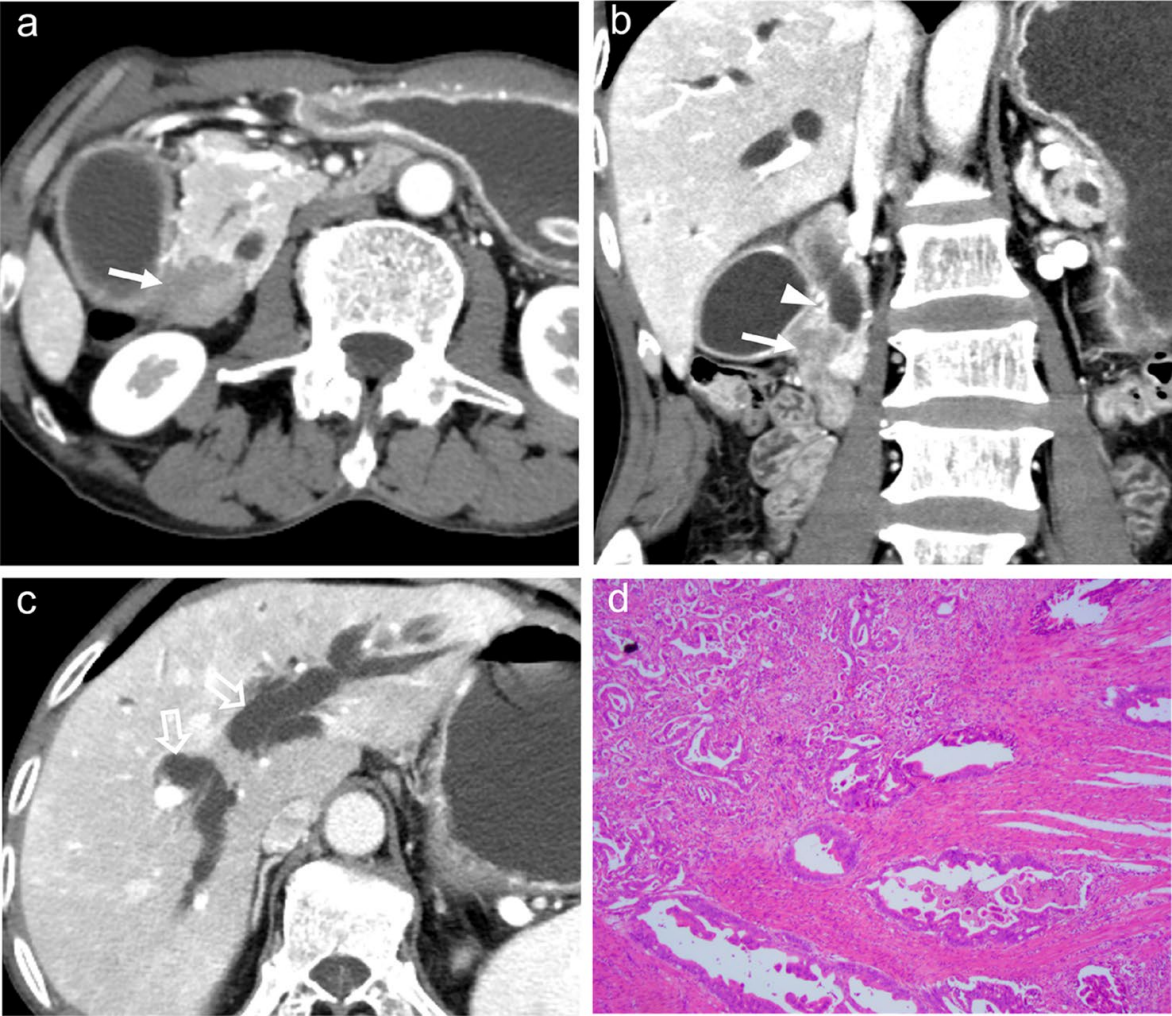
An example from a 65-year-old man who presented with jaundice. Axial (**a, c**) and coronal reconstructed images (**b**) of enhanced CT in portal venous phase show a periampullary mass with enhancement degree non-similar to that of the duodenal wall (arrows), with extrinsic growth pattern. The shape of distal CBD margin is irregular (arrowheads) accompanied by corresponding upstream bile duct dilatation (open arrow). The postoperative pathologic diagnosis of this patient was a pancreatobiliary-type periampullary adenocarcinoma (**d**)

Among the qualitative features of CT, the presence of ampulla mass, tumor growth pattern, presence of bulging ampulla, shape of the distal CBD margin, symmetry of the distal CBD lumen, presence of distal CBD wall thickening, presence of necrosis, calcification, or cystic within lesion, enhancement pattern of lesion, peak enhancement phase of lesion, main vascular involvement, and invasion of the pancreas were significantly different between the two groups in training and internal validation cohorts (*P* < 0.05) (Table 2). There were no significant differences in pattern of distal CBD narrowing, pattern of biliary dilatation, presence of IHD dilatation, degree of IHD dilatation, presence of dilated MPD, presence of peripancreatic lymph node enlargement, and presence of periampullary duodenal diverticulum. The main CT manifestations of intestinal-type periampullary adenocarcinoma and pancreatobiliary-type periampullary adenocarcinoma are shown in Figs. 2 and 3, respectively.

**Table 2:** Qualitative and quantitative evaluation CT features in each cohort

	Training and internal validation cohorts (*n* = 140)			External validation cohort (*n* = 28)		
	Pancreatobiliary type (*n* = 100)	Intestinal type (*n* = 40)	*P* value	Pancreatobiliary type (*n* = 18)	Intestinal type (*n* = 10)	*P* value
*Qualitative radiological features n (%)*						
Presence of ampullary mass	69 (69.0)	38 (95.0)	0.001	15 (83.3)	6 (60.0)	0.172
Growth pattern			< 0.001			0.129
Intrinsic	15 (15.0)	31 (77.5)		8 (44.4)	7 (70.0)	
Mixed	34 (34.0)	2 (5.0)		6 (33.3)	3 (30.0)	
Extrinsic	51 (51.0)	7 (17.5)		4 (22.2)	0 (0.0)	
Presence of bulging ampulla	42 (42.0)	34 (85.0)	< 0.001	7 (38.9)	6 (60.0)	0.283
Shape of distal CBD margin			< 0.001			0.094
Smooth	48 (48.0)	34 (85.0)		5 (27.8)	6 (60.0)	
Irregular	52 (52.0)	6 (15.0)		13 (72.2)	4 (40.0)	
Symmetry of distal CBD lumen			0.001			0.046
Symmetric	48 (48.0)	31 (77.5)		4 (22.2)	6 (60.0)	
Asymmetric	52 (52.0)	9 (22.5)		14 (77.8)	4 (40.0)	
Pattern of distal CBD narrowing			0.219			0.454
Gradual tapering	54 (54.0)	17 (42.5)		6 (33.3)	2 (20.0)	
Abrupt narrowing	46 (46.0)	23 (57.5)		12 (66.7)	8 (80.0)	
Pattern of biliary dilatation			0.856			0.649
Central	26 (26.0)	11 (27.5)		4 (22.2)	3 (30.0)	
Proportional	74 (74.0)	29 (72.5)		14 (77.8)	7 (70.0)	
Presence of IHD dilatation	95 (95.0)	36 (90.0)	0.276	18 (100.0)	10 (100. 0)	1.000
IHD dilatation extent			0.056			0.318
No dilation or mild dilation	35 (35.0)	21 (52.5)		4 (22.2)	4 (40.0)	
Moderate to severe dilation	65 (65.0)	19 (47.5)		14 (77.8)	6 (60.0)	
Presence of thickened distal CBD wall	52 (52.0)	9 (22.5)	0.001	13 (72.2)	4 (40.0)	0.094
Presence of dilated MPD	53 (53.0)	21 (52.5)	0.957	8 (44.4)	8 (80.0)	0.069
Peripancreatic lymph node enlargement	13 (13.0)	4 (10.0)	0.623	1 (5.6)	2 (20.0)	0.236
Necrosis, calcification, or cystic within lesion	18 (18.0)	1 (2.5)	0.016	2 (11.1)	0 (0)	0.308
Enhancement degree of lesion			< 0.001			0.050
Similar to the duodenum	29 (29.0)	29 (72.5)		4 (22.2)	7(70.0)	
Non-similar to the duodenum	67 (67.0)	9 (22.5)		14 (77.8)	2(20.0)	
Target-like enhancement	4 (4.0)	2 (5.0)		0 (0)	1 (10.0)	
Peak enhancement phase of lesion			< 0.001			0.015
Arterial phase	9 (9.0)	6 (15.0)		0 (0)	3 (30.0)	
Portal venous phase	51 (51.0)	31 (77.5)		5 (27.8)	4 (40.0)	
Delayed phase	40 (40.0)	3 (7.5)		13 (72.2)	3 (30.0)	
Main vascular involvement	12 (12.0)	0 (0)	0.021	8 (44.4)	8 (80.0)	0.069
Presence of periampullary duodenal diverticulum	8 (8.0)	2 (5.0)	0.534	0 (0)	0 (0)	1.000
Pancreatic involvement	64 (64.0)	8 (20.0)	< 0.001	6 (33.3)	1 (10.0)	0.172
Quantitative radiological features*						
Size of the ampullary mass (mm)	21.68 ± 9.49	19.82 ± 10.61	0.317	20.10 ± 8.51	17.43 ± 3.82	0.374
Angle of the distal CBD end (°)	85.33 ± 49.94	103.43 ± 61.01	0.105	87.14 ± 56.25	130.84 ± 60.32	0.076
Diameter of the CBD (mm)	16.86 ± 12.37	17.38 ± 22.78	0.863	17.40 ± 4.55	15.86 ± 3.23	0.370
Diameter of the MPD (mm)	3.73 ± 2.81	3.93 ± 3.28	0.716	4.18 ± 4.14	5.21 ± 2.20	0.486

Qualitative data are expressed as n (%). Chi-square or Fisher’s exact test was used for comparisons among groups. Quantitative data are expressed as mean ± SD. Students’ *t*-test was used for comparisons among groups. Statistical significance (*P* < 0.05)

CBD, common bile duct; CT, computed tomography; IHD, intrahepatic ducts; MPD, main pancreatic duct

No statistically significant differences were found between the two groups in quantitative CT characteristics in all datasets (Table 2).

### Interobserver agreement

Among the qualitative imaging variables, only main vascular involvement (*k* = 0.50) and pancreatic involvement (*k* = 0.59) showed moderate agreement, with all other variables showing good to excellent interobserver agreement (*k* = 0.64–0.95).

Of the quantitative imaging variables, the angle of the distal CBD and diameter of the CBD showed good agreement (ICC, 0.63–0.79), for the size of the ampullary mass and diameter of the MPD excellent agreement (ICC, 0.92) (Table 3).

**Table 3:** Intra- and interobserver agreement for CT imaging findings

Imaging findings	Interobserver agreement
*Qualitative imaging features*	*Weighted kappa*
Presence of ampullary mass	0.85
Growth pattern	0.77
Presence of bulging ampulla	0.81
Shape of distal CBD margin	0.84
Symmetry of distal CBD lumen	0.64
Pattern of distal CBD narrowing	0.90
Pattern of biliary dilatation	0.82
Presence of IHD dilatation	0.89
IHD dilatation extent	0.92
Presence of thickened distal CBD wall	0.80
Presence of dilated MPD	0.91
Peripancreatic lymph node enlargement	0.94
Necrosis, calcification, or cystic within lesion	0.71
Enhancement degree of lesion	0.79
Peak enhancement phase of lesion	0.78
Main vascular involvement	0.50
Presence of periampullary duodenal diverticulum	0.95
Pancreatic involvement	0.59
*Quantitative imaging variables*	*ICC*
Size of the ampullary mass (mm)	0.92
Angle of the distal CBD end (°)	0.63
Diameter of the CBD (mm)	0.79
Diameter of the MPD (mm)	0.92

ICC, intraclass correlation coefficient

### Development and validation of ML classifiers

**Fig. 4:**
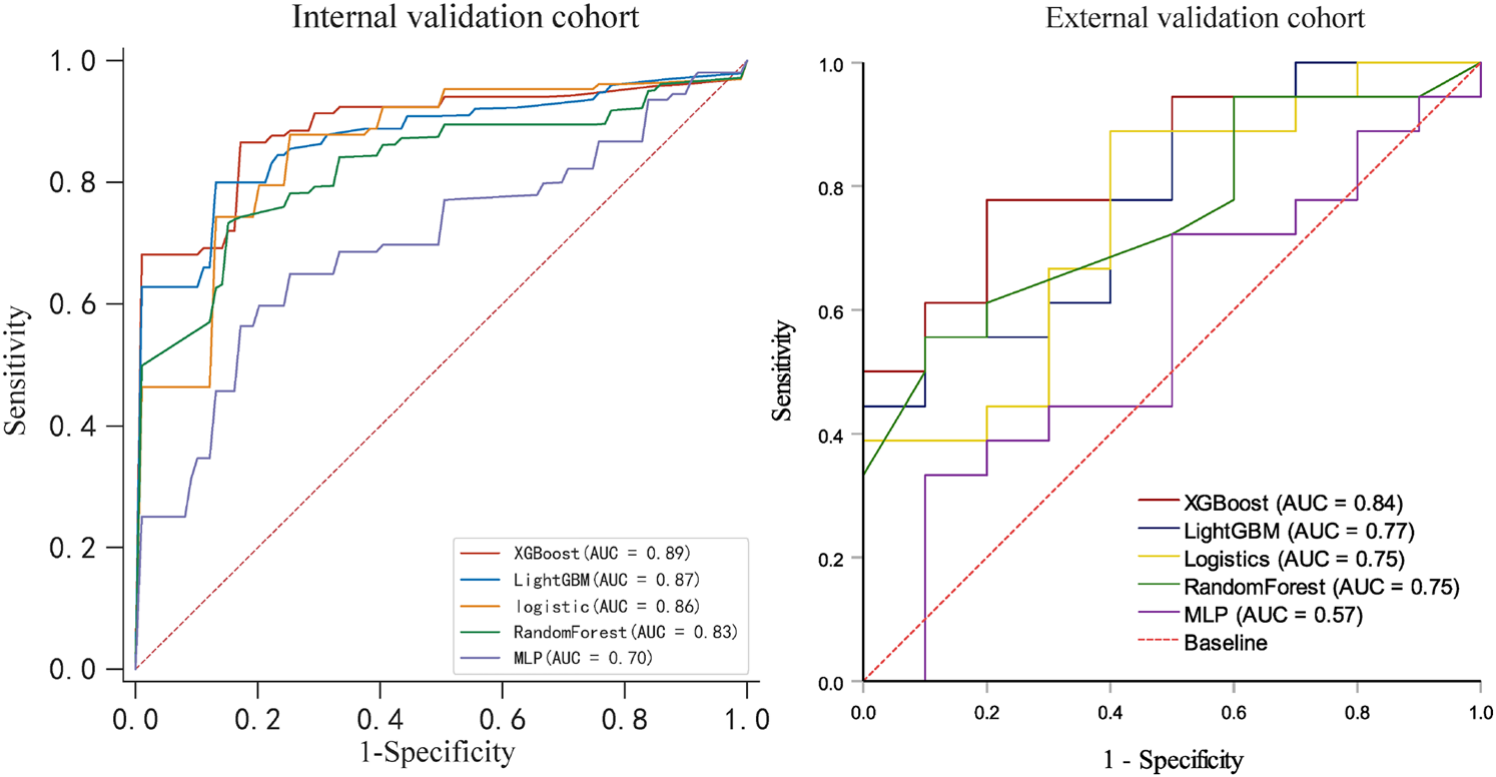
ROC curves for five machine learning classifiers based on imaging and clinical features, in the internal validation cohort and external validation cohort, respectively. AUC, area under curve; LightGBM, light gradient boosting; MLP, multi-layer perceptron; XGBoost, eXtreme Gradient Boosting

We selected qualitative CT and clinical features to construct models based on LightGBM, MLP, XGBoost, random forest, and logistic regression. The hyperparameters of all classifiers are shown in Supplement Table 6. The ROC of the prediction models based on different classifiers in the internal validation cohort and external validation cohort are shown in Fig. 4 and Table 4. Except for MLP, all the other four classifiers performed well in each dataset. The XGBoost shows the highest AUC value in the training set, the internal validation set, and the external validation set.

**Table 4:** Diagnostic performance of different machine learning classifiers in internal validation cohort and external validation cohort

Model	AUC	Accuracy	Sensitivity	Specificity	PPV	NPV	F1 score
*Internal validation cohort*							
XGBoost	0.89 (0.76–1.00)	0.86	0.83	0.97	0.94	0.72	0.87
LightGBM	0.87 (0.74–0.99)	0.84	0.83	0.91	0.94	0.69	0.88
Logistic	0.86 (0.69–1.00)	0.76	0.82	0.86	0.94	0.48	0.88
RandomForest	0.83 (0.66–0.98)	0.79	0.82	0.85	0.92	0.58	0.86
MLP	0.70 (0.50–0.90)	0.63	0.73	0.75	0.82	0.44	0.77
*External validation cohort*							
XGBoost	0.84 (0.69–0.99)	0.79	1.00	0.50	0.64	1.00	0.87
LightGBM	0.77 (0.60–0.95)	0.61	0.61	0.60	0.73	0.46	0.67
Logistic	0.75 (0.56–0.94)	0.68	0.67	0.70	0.80	0.54	0.73
RandomForest	0.75 (0.56–0.93)	0.71	0.78	0.60	0.78	0.60	0.78
MLP	0.57 (0.33–0.79)	0.50	0.44	0.60	0.67	0.38	0.53

AUC, area under curve; LightGBM, light gradient boosting; MLP, multi-layer perceptron; NPV, negative predictive value; PPV, positive predictive value; XGBoost, eXtreme Gradient Boosting

Compared to other classifiers, the XGBoost shows the highest AUC values in the training set, the internal validation set, and the external validation set. This is statistically different in the internal validation set (Supplement Table 1) but not significant in the external validation set (*P* > 0.05) (Supplement Table 2).

Considering that traditional logistic regression model also have good diagnostic performance, we additionally performed univariate and multivariate regression analysis (Supplement Table 5). Moreover, nomogram was constructed based on the logistic regression model (Supplement Fig. 1).

### Visualization of feature importance for the best classifier

**Fig. 5:**
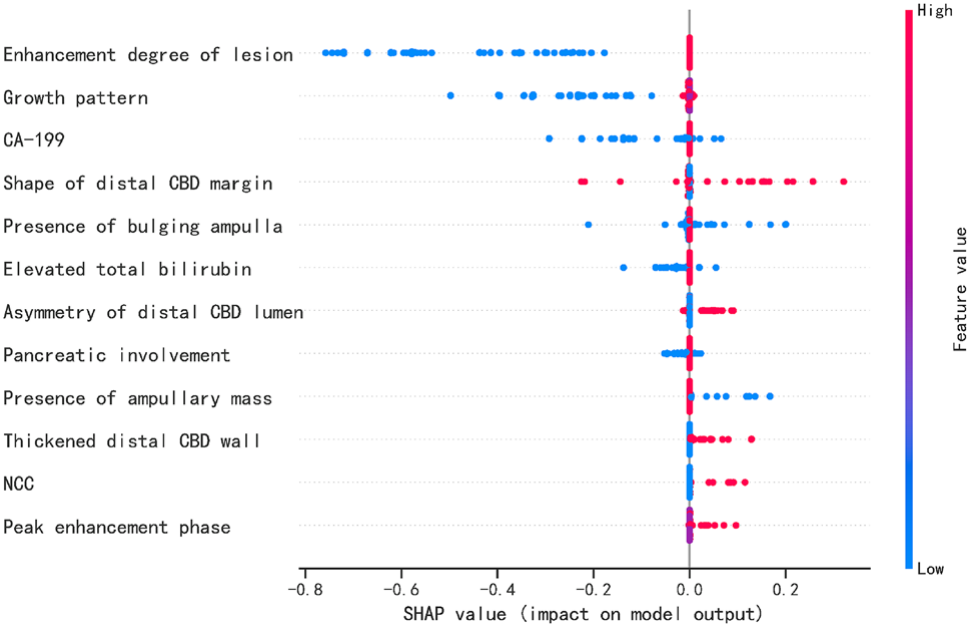
Summary plot of the importance of features in XGBoost classifier. *Y*-axis represents the importance of the features, in descending order. *x*-axis represents the contribution, where > 0 is a positive contribution and < 0 is a negative contribution. The color of the scatter indicates whether the feature is high (red) or low (blue). CBD, common bile duct; MPD, main pancreatic duct; NCC, presence of necrosis, calcification, or cystic within lesion

To visually explain the features included in the XGBoost, we used SHAP to explain the role of these features in differentiating pancreatobiliary and intestinal-type periampullary adenocarcinomas in the model (Fig. 5). The SHAP values (*x*-axis) are a uniform quantification of the impact of the features included in the model, and the impact on the results is plotted with two colored dots. The red dots represent high-risk values, and the blue ones represent low-risk values. The top 10 features were enhancement degree, growth pattern, elevated CA19-9 levels, shape of distal CBD margin, presence of bulging ampulla, elevated total bilirubin, asymmetry of distal CBD lumen, pancreatic involvement, presence of ampullary mass, and presence of thickened distal CBD wall.

### Calibration curve and DCA of the best classifier in external validation

In external validation, the calibration curve of XGBoost (Supplement Fig. 2A) showed that the predicted value was close to the actual value, which indicated that the model was well calibrated; the DCA of XGBoost (Supplement Fig. 2B) suggestted that net clinical efficacy was obtained in practical applications.

### Prognostic value of the best classifier

**Fig. 6:**
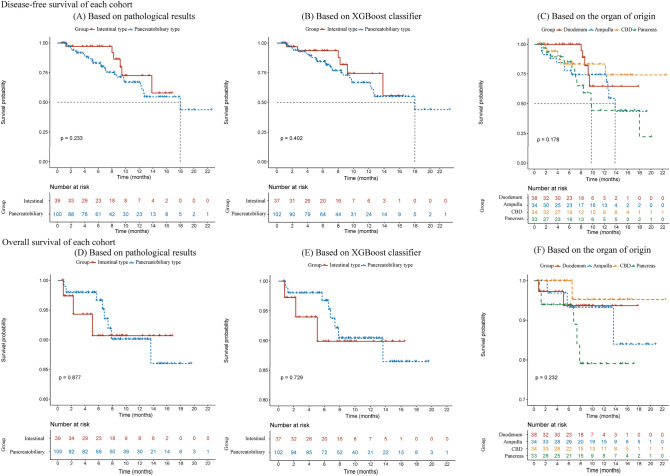
Kaplan–Meier curves of disease-free survival (DFS) and overall survival (OS) rates for pancreatobiliary- and intestinal-type ampullary carcinomas (**A, D**). Kaplan–Meier curves of DFS and OS rates for the model-predicted pancreaticobiliary and intestinal types differentiated based on the combined model (**B, E**). Kaplan–Meier curves of disease-free survival (DFS) and overall survival (OS) rates based on ampullary carcinomas of four anatomic organ origins (**C, F**). There was no significant difference between the groups (log-rank test, *P* > 0.05). CBD, common bile duct

Our results showed a trend of higher DFS and OS for the intestinal periampullary adenocarcinoma than for the pancreatobiliary periampullary adenocarcinoma in our cohort, but there were no statistical differences (*P* > 0.05). We divided the patients into model-predicted intestinal- and pancreatobiliary-type periampullary adenocarcinomas based on the combined model, and the results were similar to the actual pathology-based grouping (*P* > 0.05). In addition, no statistical differences in DFS and OS were found in subgroups based on organ origin (Fig. 6).

## Discussion

The present study constructed diagnostic models incorporated CT imaging and clinical features for differentiating pancreatobiliary- and intestinal-type periampullary adenocarcinomas using various ML classifiers. The results showed that all classifiers except MLP showed good performance. Consequently, the XGBoost classifier exhibited high effectiveness on the training (AUC = 0.98), internal validation (AUC = 0.89), and external validation (AUC = 0.84) cohorts. Based on our best ML classifier, enhancement degree, growth pattern of lesion, and CA 19-9 were the predictors with the strongest feature importance.

In the internal validation, our ML models have similar performance except for MLP, but XGBoost outperforms the other models significantly in the external validation. Generally, XGBoost's performance is outperforming other models such as LR, RF, and MLP in the majority of prediction tasks. Firstly, XGBoost's gradient enhancement framework iteratively refines the process of predictive model building, effectively learning from previous mistakes and thus improving the accuracy over time [25]. XGBoost also allows for a depth-first approach through built-in regularization mechanisms and a unique tree pruning method, which helps to reduce overfitting [26]. Also, XGBoost includes built-in cross-validation at each iteration, which helps optimize model tuning. However, we used LightGBM as well, which is also a gradient enhancement framework, and in some scenarios, it stands out for its speed and efficiency, especially when working with large datasets. But XGBoost outperforms it in the external validation, probably due to the fact that XGBoost has the ability to perform the prediction task more efficiently with more complex tree pruning and effective use of parallel processing. However, a larger cohort for validation is still required to show the applicability of the XGBoost model.

Laboratory findings, such as CA19-9 and total bilirubin, have been applied in clinical work for the differentiation of benign and malignant ampullary lesions [20, 21]. However, no previous literature has evaluated the differences between pancreatobiliary- and intestinal-type periampullary adenocarcinomas regarding CA19-9 and total serum bilirubin level. Our study showed that pancreatobiliary periampullary adenocarcinoma patients had a higher proportion of elevated total bilirubin and CA19-9 than intestinal periampullary adenocarcinoma patients. Moreover, CA19-9 was the 3rd most important predictor in the best classifier we developed. The higher levels of bilirubin and CA19-9 observed in patients with peripancreatobiliary-type adenocarcinomas than in intestinal-type adenocarcinomas may be due to anatomical and biological differences between these subtypes. The anatomical location of tumors of epithelial origin in the pancreaticobiliary duct is more likely to result in bile duct obstruction, which may lead to bilirubin accumulation in the blood [8]. In addition, the biological behavior of these tumors typically results in a more pronounced expression of CA19-9, which is particularly relevant to pancreatic ductal carcinomas and may reflect a greater tumor load or a more aggressive tumor phenotype. Two recent studies focusing on ampullary adenocarcinoma reported more elevated total bilirubin and elevated CA19-9 in pancreatobiliary type than in intestinal type [13, 27], which partially supports our results.

Several previous studies have examined the value of image enhancement features in distinguishing between pancreatobiliary-type and intestinal-type adenocarcinomas [18, 28, 29]. Two MRI studies reported that pancreatobiliary periampullary adenocarcinoma more frequently exhibited progressive enhancement compared with intestinal periampullary adenocarcinoma [18, 28]. This difference in enhancement can be attributed to the higher prevalence of desmoplastic stroma in pancreatobiliary-type adenocarcinomas, making them more prone to show progressive enhancement [30]. However, Gündüz et al. [29] found no distinction in CT enhancement patterns between pancreaticobiliary and intestinal subtypes of periampullary pancreatic ductal adenocarcinoma. In our study, although there were differences in enhancement patterns between the two groups, these distinctions played a minor role in the XGBoost classifier. Furthermore, we referred to studies that identified benign and malignant ampullary lesions [21] and additionally evaluated the enhancement discrepancy of the lesion and the adjacent normal duodenal wall, specifically in the portal phase. We categorized the enhancement discrepancy into three groups: similar or dissimilar to the duodenal enhancement pattern, or target-like enhancement. Approximately 73% of intestinal periampullary adenocarcinomas exhibited similarity to duodenal enhancement, while about 67% of pancreatobiliary periampullary adenocarcinomas showed the opposite pattern, with a similar proportion of target-like enhancement between the two groups. Notably, this particular feature emerged as the most significant factor in XGBoost classifier.

Our research findings indicate notable distinctions in the growth patterns between intestinal-type and pancreatobiliary periampullary adenocarcinomas. Specifically, intestinal-type tumors exhibit a tendency to grow intraluminally, while pancreaticobiliary tumors display the highest percentage of extraluminal growth. This observation implies that the proximity of the tumor center to the duodenal papilla may be more likely to be diagnosed as an intestinal-type adenocarcinoma. Additionally, the pancreatobiliary-type adenocarcinomas demonstrate a higher proportion of mixed growth patterns compared to the intestinal type, further supporting the notion of its more aggressive nature [31, 32].

Among the CT qualitative features, our study revealed that pancreatobiliary periampullary adenocarcinomas more frequently exhibited irregular distal CBD margin, ranking as the 4th most important feature in the XGBoost classifier. Nalbant et al. [28]found that irregular narrowing in the distal margin of the CBD is suggestive of pancreatobiliary periampullary adenocarcinoma in MRI, while some studies have not found the positive value of this feature [18, 23]. Our results are more supportive of the former, as irregularity of the distal margins of the CBD seems to represent a highly aggressive tumor. The pancreatobiliary periampullary adenocarcinoma displays a cell phenotype similar to that of pancreatic ductal or extrahepatic bile duct carcinomas [33]. It is more prone to periductal infiltrative growth, manifesting as biliary ductal stricture with scar-like fibrosis. The pathologic basis may be due to associated stromal desmoplasia [34, 35]. Furthermore, in agreement with a previous study [21], the interobserver agreement for this metric in our study was excellent (*k* > 0.80). Therefore, we conclude that the shape of the distal margin of the CBD is an important CT feature for differentiating pancreaticobiliary and intestinal peripancreatic adenocarcinoma. In addition, previous studies have reported that the gastroduodenal artery involvement and lymph node enlargement were significantly associated with pancreatobiliary-type adenocarcinomas [28, 29], while oval filling defects of the distal bile duct were significantly associated with intestinal-type adenocarcinomas [23, 28]. These metrics were not given sufficient weight in our model.

In previous studies, quantitative parameters such as the mass size, the diameter of CBD, and the diameter of MPD have not been reported to differ in intestinal-type and pancreaticobiliary-type adenocarcinomas [18, 28, 29]. It is controversial whether the size of the mass is a prognostic factor for periampullary adenocarcinoma [7, 36, 37]. Likewise, our results did not find a contribution from the above-mentioned indicators. We were the first study to apply the distal CBD angle to the comparison of pancreatobiliary- and intestinal-type periampullary adenocarcinomas, although non-significant results were reported. Lee et al. [21] reported that this feature revealed a statistical difference between benign and malignant ampullary stenosis, but the statistical difference disappeared after multivariate analysis. Therefore, whether these quantitative parameters can be biomarkers to distinguish different subtypes of periampullary adenocarcinoma still needs further validation.

Our results regarding prognosis need to be interpreted with caution. Previous findings have shown that the prognosis of the intestinal type was better than that of the pancreatobiliary type [16]. Although our results showed a trend of higher DFS and OS for the intestinal type than for the pancreaticobiliary type, there was no statistical difference. This may be due to our short follow-up period, which limits our observation of long-term prognosis. In addition, since our study only included patients who could undergo curative surgery, this may have narrowed the difference in prognosis between the two. Nevertheless, the prognostic performance of our model-based subgroups was similar to that of the pathology-based subgroups, suggesting the potential for models of imaging combined with clinical features as an additional method to predict prognosis to guide clinical practice.

It is important to mention that our ML classifier cannot replace endoscopic biopsy examination. First, our current study did not analyze the diagnostic accuracy of ML model and endoscopic biopsy comparatively. We found that a sizeable proportion of patients in our study did not undergo preoperative endoscopic biopsy. Second, both CT examination and endoscopic biopsy are recommended examinations for patients presenting with clinical suspicion of ampullary neoplasm. Although endoscopic biopsies of ampullary adenocarcinoma have some disadvantages, such as low diagnostic accuracy and high false-negative rate (20–40%) [38]. However, the complex histologic information obtained from endoscopic biopsies remains crucial for developing accurate treatment strategies. Finally, although our study develops a ML diagnostic model based on imaging and clinical parameters that can satisfactorily differentiate subtypes of ampullary adenocarcinoma and was validated in an external validation dataset. However, further validation in larger samples is still needed. Therefore, as a noninvasive predictive tool, our ML model may help clinicians determine which patients are more in need of endoscopic biopsy, thus avoiding unnecessary waste of medical resources and alleviating patient suffering. Although promising, we suggest that our model should be incorporated into a broader diagnostic framework rather than replacing endoscopic biopsy altogether.

This study has some limitations. Firstly, this was a retrospective study that included patients with surgical pathology results. Hence, our model may not generalize to patients with advanced cancer who cannot undergo surgery. Secondly, our sample size was small; however, we used multiple ML classifiers to find the best hyperparameters in the training cohort along with internal fivefold cross-validation. The performance of the ML classifiers was fully validated in both the internal validation cohort and the external validation cohort to ensure reproducibility. Future external validation cohorts with larger samples are still needed to further validate the robustness of the classifiers.

In conclusion, we developed and validated a combined model based on measurable qualitative CT and clinical features. In our study, the combined model using XGBoost performed best in distinguishing the subtypes of periampullary adenocarcinomas, which may help to improve the clinical treatment management strategy of periampullary adenocarcinomas.

### Supplementary Information

Below is the link to the electronic supplementary material.

Supplementary file1 (DOCX 199 KB)
