# Supplementary material for: Machine learning for differentiating between pancreatobiliary-type and intestinal-type periampullary carcinomas based on CT imaging and clinical findings
Source: Abdom Radiol (NY). 2024 Jan 18;49(3):748–61. doi: 10.1007/s00261-023-04151-1 (PMC10909762; doi:10.1007/s00261-023-04151-1)
Supplement: Supplementary file 1 — Supplementary file1 (DOCX 199 KB) [file 261_2023_4151_MOESM1_ESM.docx]

**SUPPLEMENTARY INFORMATION**

**Machine learning for differentiating between pancreatobiliary-type and intestinal-type periampullary adenocarcinomas based on CT imaging and clinical findings.**

**Chen et al.**

**Supplementary Materials**

**• Supplementary Tables**

**• Supplementary Figures**

**Supplementary Tables**

**Supplementary Table 1. P-value of the Delong test for the internal validation set.**

| Classifiers | XGB | LightGBM | Logistic | RandomForest | MLP |
| --- | --- | --- | --- | --- | --- |
| XGB | NA | 0.04 | 0.04 | 0.09 | 0.05 |
| LightGBM | 0.04 | NA | 0.5 | 0.39 | 0.66 |
| Logistic | 0.04 | 0.5 | NA | 0.18 | 0.29 |
| RandomForest | 0.09 | 0.39 | 0.18 | NA | 0.52 |
| MLP | 0.05 | 0.66 | 0.29 | 0.52 | NA |

Abbreviations: LightGBM, light gradient boosting; MLP, multi-layer perceptron; XGBoost, eXtreme Gradient Boosting.

**Supplementary Table 2. P-value of the Delong test for the external validation set.**

| Classifiers | XGBoost | LightGBM | Logistic | RandomForest | MLP |
| --- | --- | --- | --- | --- | --- |
| XGBoost |  | 0.068 | 0.955 | 0.89 | 0.145 |
| LightGBM | 0.068 |  | 0.172 | 0.103 | 0.541 |
| Logistic | 0.955 | 0.172 |  | 0.839 | 0.158 |
| RandomForest | 0.89 | 0.103 | 0.839 |  | 0.218 |
| MLP | 0.145 | 0.541 | 0.158 | 0.218 |  |

Abbreviations: LightGBM, light gradient boosting; MLP, multi-layer perceptron; XGBoost, eXtreme Gradient Boosting.

**Supplementary Table 3. Z-value of the delong test for the internal validation set.**

| Classifiers | XGB | LightGBM | Logistic | RandomForest | MLP |
| --- | --- | --- | --- | --- | --- |
| XGB | NA | 3.01 | 2.91 | 2.59 | 2.16 |
| LightGBM | 3.01 | NA | 1.18 | 1.36 | 0.55 |
| Logistic | 2.91 | 1.18 | NA | 1.45 | 1.54 |
| RandomForest | 2.59 | 1.36 | 1.45 | NA | 0.87 |
| MLP | 2.16 | 0.55 | 1.54 | 0.87 | NA |

Abbreviations: LightGBM, light gradient boosting; MLP, multi-layer perceptron; XGBoost, eXtreme Gradient Boosting.

**Supplementary Table 4. Z-value of the Delong test for the external validation set.**

| Classifiers | XGB | LightGBM | Logistic | RandomForest | MLP |
| --- | --- | --- | --- | --- | --- |
| XGBoost |  | 1.822 | 0.056 | 0.139 | 1.458 |
| LightGBM | 1.822 |  | 1.365 | 1.63 | 0.611 |
| Logistic | 0.056 | 1.365 |  | 0.203 | 1.41 |
| RandomForest | 0.139 | 1.63 | 0.203 |  | 1.232 |
| MLP | 1.458 | 0.611 | 1.41 | 1.232 |  |

Abbreviations: LightGBM, light gradient boosting; MLP, multi-layer perceptron; XGBoost, eXtreme Gradient Boosting.

**Supplementary Table 5. Univariable and Multivariable Logistic Regression Analysis of the**

**Clinical and imaging variables in the training set and the internal validation set.**

| Variables | Univariable | | Multivariable | |
| --- | --- | --- | --- | --- |
|  | OR (95% CI) | p value | OR (95% CI) | p value |
| Elevated total bilirubin | 2.45 [1.14,5.24] | 0.02 |  |  |
| Elevated CA-199 | 2.72 [1.28,5.78] | 0.01 |  |  |
| Presence of ampullary mass | 0.12 [0.03,0.52] | < 0.001 | 0.21 [0.03,1.07] | 0.09 |
| Growth pattern |  |  |  |  |
| Mixed | 15.06 [5.53,41.01] | < 0.001 | 8.97 [3.06,29.40] | < 0.001 |
| Extrinsic | 35.13 [7.43,166.13] | < 0.001 | 19.73 [4.66,138.18] | < 0.001 |
| Presence of bulging ampulla | 0.13 [0.05,0.33] | < 0.001 |  |  |
| Shape of distal CBD margin | 6.14 [2.37,15.91] | < 0.001 | 2.71 [0.87,9.26] | 0.09 |
| Symmetry of distal CBD lumen | 3.73 [1.61,8.64] | < 0.001 |  |  |
| Presence of thickened distal CBD wall | 3.73 [1.61,8.64] | < 0.001 |  |  |
| Presence of necrosis, calcification, cystic within lesion | 8.56 [1.10,66.46] | 0.04 |  |  |
| Enhancement degree of lesion |  |  |  |  |
| Non-similar to the duodenum | 7.44 [3.13,17.69] | < 0.001 | 4.48 [1.56,13.94] | 0.01 |
| Target-like enhancement | 2.00 [0.34,11.79] | 0.44 | 3.68 [0.42,37.25] | 0.24 |
| Peak enhancement phase of lesion |  |  |  |  |
| Portal venous phase | 1.10 [0.36,3.38] | 0.87 |  |  |
| Delayed phase | 8.89 [1.86,42.44] | 0.01 |  |  |
| Pancreatic involvement | 7.11 [2.96,17.07] | 0.00 |  |  |

Abbreviations: CBD, common bile duct; CI, confidence interval; OR, odds ratio.

**Supplementary Table 6. Hyperparameters for All** **Classifiers.**

| **Classifiers** | **Hyperparameters** |
| --- | --- |
| LightGBM | boosting_type = gbdt |
|  | learning_rate = 0.001 |
|  | max_depth = 20 |
|  | n_estimators = 5 |
|  | num_leaves = 5 |
|  |  |
| Random Forest | criterion = gini |
|  | max_depth = None |
|  | min_impurity_decrease = 0.0 |
|  | n_estimators = 20 |
|  |  |
| XGBoost | reg_lambda = 0.5 |
|  | min_child_weight = 6 |
|  | max_depth = 4 |
|  | learning_rate = 0.1 |
|  |  |
| MLP | activation = relu |
|  | hidden_layer_sizes = (20, 10) |
|  | max_iter = 20 |
|  |  |
| Logistic Regression | tol = 0.0001 |
|  | penalty = l2 |
|  | max_iter = 100 |
|  | C = 0.09999999999999999 |

Abbreviations: LightGBM, light gradient boosting; MLP, multi-layer perceptron; XGBoost, eXtreme Gradient Boosting.

**Supplementary Figures**


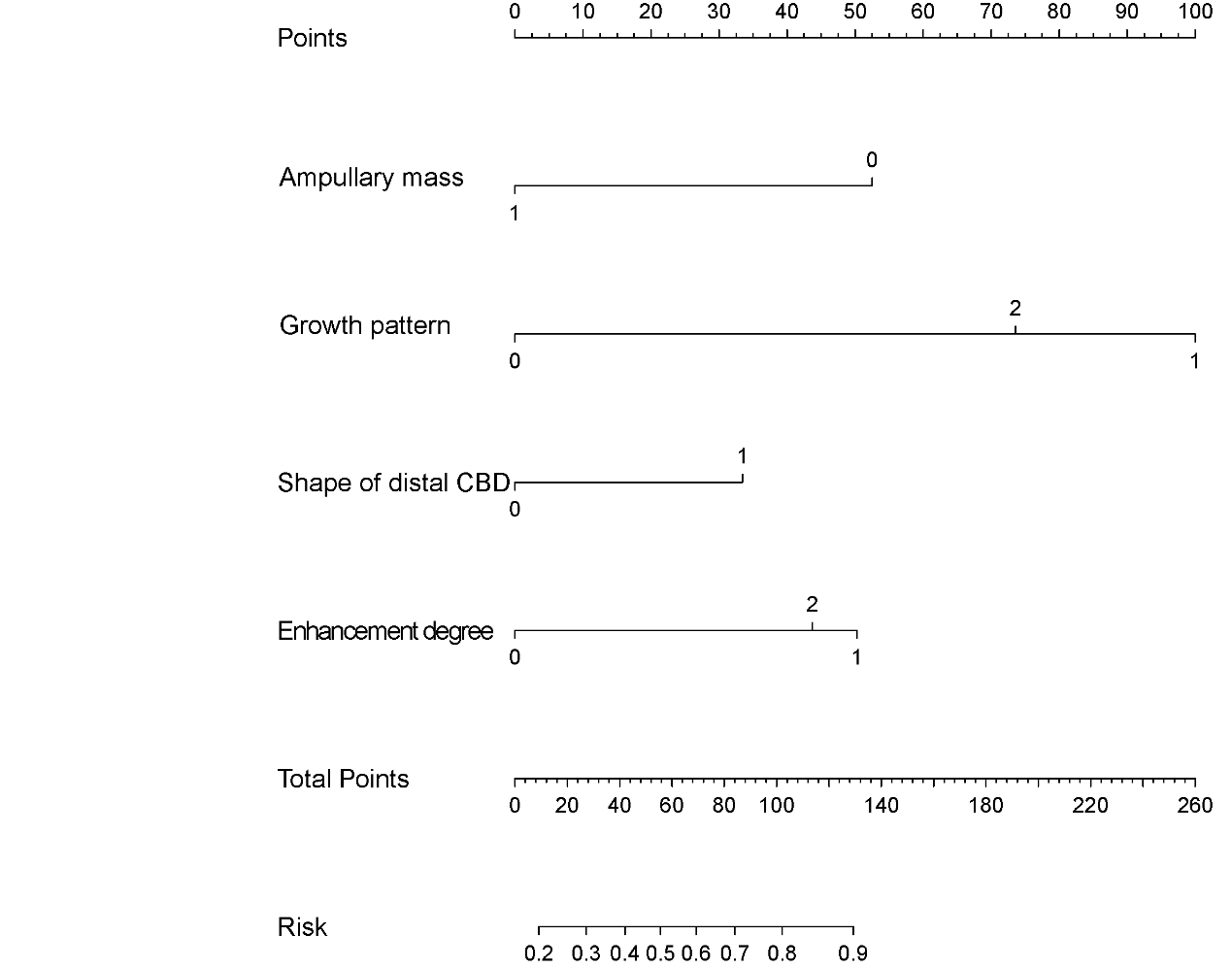


**Supplementary Figure 1. Nomogram of the imaging model for predicting the pancreatobiliary type periampullary adenocarcinoma risk using Logistic regression method.**

Abbreviations: CBD, common bile duct.

**
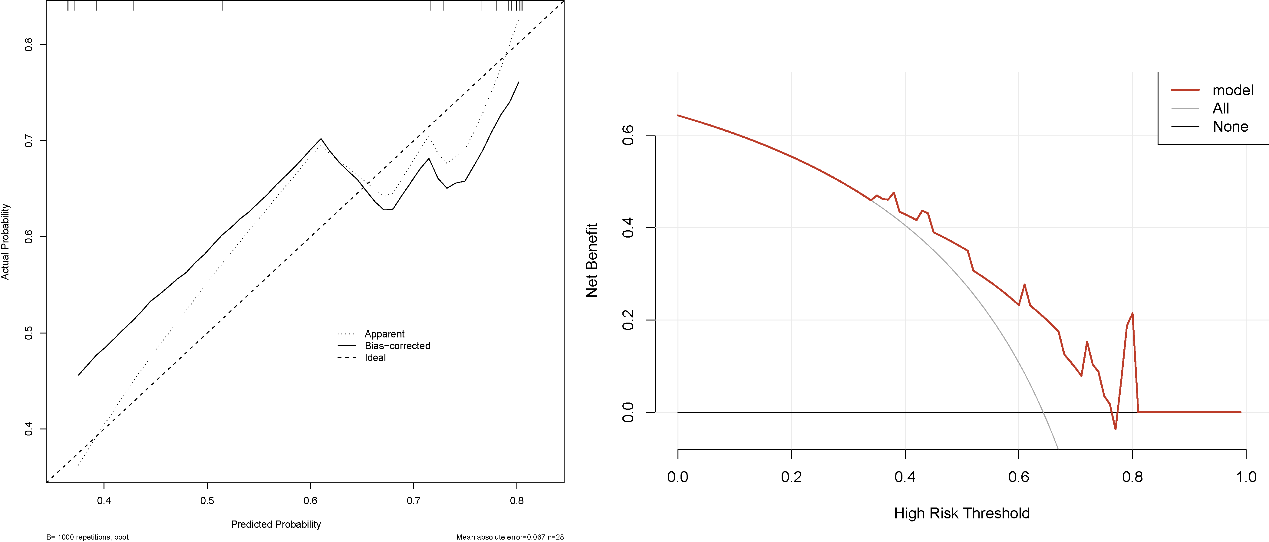
**

**Supplementary Figure 2. The plotted calibration curves (A) and DCA curves (B) for XGBoost classifier in the external validation set.**
